# High-resolution patterns and inequalities in ambient fine particle mass (PM_2.5_) and black carbon (BC) in the Greater Accra Metropolis, Ghana

**DOI:** 10.1016/j.scitotenv.2023.162582

**Published:** 2023-06-01

**Authors:** Abosede S. Alli, Sierra N. Clark, Jiayuan Wang, James Bennett, Allison F. Hughes, Majid Ezzati, Michael Brauer, James Nimo, Josephine Bedford-Moses, Solomon Baah, Alicia Cavanaugh, Samuel Agyei-Mensah, George Owusu, Jill Baumgartner, Raphael E. Arku

**Affiliations:** a Department of Environmental Health Sciences, School of Public Health and Health Sciences, University of Massachusetts, Amherst, USA; b Department of Epidemiology and Biostatistics, School of Public Health, Imperial College London, London, UK; c MRC Centre for Environment and Health, School of Public Health, Imperial College London, London, UK; d Department of Physics, University of Ghana, Accra, Ghana; e Regional Institute for Population Studies, University of Ghana, Accra, Ghana; f School of Population and Public Health, The University of British Columbia, Vancouver, Canada; g Department of Geography, McGill University, Montreal, Canada; h Department of Geography and Resource Development, University of Ghana, Accra, Ghana; i Institute of Statistical, Social & Economic Research, University of Ghana, Accra, Ghana; j Institute for Health and Social Policy, McGill University, Montreal, Canada; k Department of Epidemiology, Biostatistics, and Occupational Health, McGill University, Montreal, Canada

**Keywords:** Land use regression, Air pollution, Fine particulate matter (PM_2.5_), Black carbon, Socio-economic status, Sub-Saharan Africa, Ghana

## Abstract

Growing cities in sub-Saharan Africa (SSA) experience high levels of ambient air pollution. However, sparse long-term city-wide air pollution exposure data limits policy mitigation efforts and assessment of the health and climate effects. In the first study of its kind in West Africa, we developed high resolution spatiotemporal land use regression (LUR) models to map fine particulate matter (PM_2.5_) and black carbon (BC) concentrations in the Greater Accra Metropolitan Area (GAMA), one of the fastest sprawling metropolises in SSA. We conducted a one-year measurement campaign covering 146 sites and combined these data with geospatial and meteorological predictors to develop separate Harmattan and non-Harmattan season PM_2.5_ and BC models at 100 m resolution. The final models were selected with a forward stepwise procedure and performance was evaluated with 10-fold cross-validation. Model predictions were overlayed with the most recent census data to estimate the population distribution of exposure and socioeconomic inequalities in exposure at the census enumeration area level. The fixed effects components of the models explained 48–69 % and 63–71 % of the variance in PM_2.5_ and BC concentrations, respectively. Spatial variables related to road traffic and vegetation explained the most variability in the non-Harmattan models, while temporal variables were dominant in the Harmattan models. The entire GAMA population is exposed to PM_2.5_ levels above the World Health Organization guideline, including even the Interim Target 3 (15 μg/m^3^), with the highest exposures in poorer neighborhoods. The models can be used to support air pollution mitigation policies, health, and climate impact assessments. The measurement and modelling approach used in this study can be adapted to other African cities to bridge the air pollution data gap in the region.

## Highlights

• Few long-term city-wide air pollution exposure data exists in growing SSA cities.
• We provide the first space-time city-wide mapping of PM_2.5_ and BC in West Africa.
• Monitoring data at 146 sites were combined with GIS, weather, and census variables.
• Models explained 48–69 % and 63–71 % of the variance in PM_2.5_ and BC, respectively.
• PM_2.5_ levels were above the WHO IT-3 with higher exposures in poorer neighborhoods.

## Graphical abstract

**Figure:**
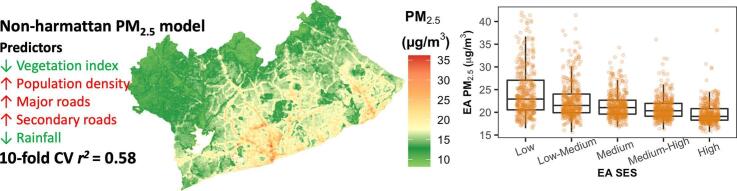

## 1 Introduction

Ambient air pollution is a major environmental risk factor for death and ill-health globally (Anenberg et al., 2019; Cohen et al., 2017). Most of the estimated global deaths attributed to ambient air pollution occur in low-and middle-income countries (LMICs) (Cohen et al., 2017; World Health Organization, 2016). Emerging evidence from mostly short-term measurement studies (≤24-hour) indicates that ambient air pollution in sub-Saharan African (SSA) cities are among the highest in the world and substantially exceed the World Health Organization (WHO) health-based guidelines (Katoto et al., 2019). However, limited robust long-term air pollution data hinders mitigation efforts and quantification of the health and climate effects (Awokola et al., 2020; Malings et al., 2020; Coker and Kizito, 2018) in growing SSA cities. As SSA cities expand, systematic and long-term tracking of the sources and variations in air quality in fine spatial resolution can facilitate specific policy interventions that are socio-culturally relevant to SSA settings (Katoto et al., 2019; Coker and Kizito, 2018). Further, reliable long-term spatial and time-resolved data for addressing air pollution in SSA cities will contribute to the global fight against climate change, with significant health benefits for a region where an estimated 1.4 billion people will live by year 2050 (United Nations, 2022).

Patterns and inequalities in air pollution exposure in SSA cities are influenced by a complex mix of local (e.g., transportation, biomass use, informal industries, etc.) and regional sources (crustal dust from regional dust storms) (Petkova et al., 2013). Thus, any air quality management in a changing global climate in SSA will require detailed mapping of pollutant concentration over space and time. Given the high cost of establishing and maintaining ambient air quality monitoring networks, it is imperative to explore high resolution space-time mapping approaches to supplement the scant monitoring data in this low-resource and data-poor region (Coker and Kizito, 2018). Though widely employed in European (Hoek et al., 2011; Eeftens et al., 2012; Beelen et al., 2013), North American (Henderson et al., 2007; Moore et al., 2007; Novotny et al., 2011), and Asian (Saraswat et al., 2013; Lee et al., 2017; Liu et al., 2019) cities, fine space-time air pollution data are scarce in SSA cities where poor air quality presents a major health, economic, and climate threat (Coker and Kizito, 2018). Moreover, the sources and influence of socio-economic indices on spatial patterns of air pollution may differ considerably from those in high-income countries (Coker and Kizito, 2018) and will in turn require specific mitigation approaches.

This study developed land use regression (LUR) models to predict and map fine-scale spatiotemporal variations in ambient particulate matter pollution (PM_2.5_) and black carbon (BC) in one of the largest metropolises in West Africa. LUR modelling offers a cost-effective approach for capturing high-resolution, within-city variability in air pollution (Beelen et al., 2013; Saraswat et al., 2013; Saucy et al., 2018). To our knowledge, LUR technique has only been applied to model PM_2.5_ in four SSA cities (Saucy et al., 2018; Tularam et al., 2020; Abera et al., 2020; Coker et al., 2021), and none is the West African sub-region. We are not aware of any LUR studies of BC, a combustion-related component of PM_2.5_ and an important climate change pollutant. We integrated field data from a large-scale air pollution measurement campaign conducted in the Greater Accra Metropolis of Ghana (Alli et al., 2021), with meteorological and geospatial data. The final models were used to assess the distribution of population exposure to predicted PM_2.5_ and BC levels and inequalities in exposure in relation to area level socio-economic status.

## 2 Methods

### 2.1 Study location

This study was conducted in the Greater Accra Metropolitan Area (GAMA), Ghana's hub of administrative, industrial, and economic activities (Addae and Oppelt, 2019). The GAMA spans ~1500 km^2^ and comprises of 13 districts, including the Accra Metropolitan Area (AMA) at its center (~1.66 million residents) and the port city of Tema (600,000 residents) to the east (GSS, 2012). Though the metropolis is expanding rapidly, huge gaps exist between the demand and provision of adequate infrastructure (e.g. clean roads and household energy) for its urban residents (Addae and Oppelt, 2019; Odonkor and Mahami, 2020). Major sources of air pollution in the GAMA include road traffic, industrial emissions, household biomass use, and seasonal regional dust storms (Zhou et al., 2013). Other sources unique to the GAMA include open burning of trash and solid waste, especially in low-income neighborhoods (Zhou et al., 2013). The GAMA's climate is characterized by the rainy (May–October) season, which is dominated by primary emissions from local sources; and the dry and dusty Harmattan season (November–February) characterized by north-easterly trade winds from the Sahara Desert along with changes in local meteorology (e.g., no rainfall, lower relative humidity, and wind speed) that may create slower vertical mixing and result in manifold increases in air pollution levels (Alli et al., 2021; Dionisio et al., 2010a; Wang et al., 2021).

### 2.2 Data

#### 2.2.1 Ambient PM_2.5_ and BC measurement

Between April 2019 and June 2020, we measured gravimetric (filter-based) and continuous PM_2.5_ concentrations at 146 locations comprising of ‘fixed’ (~1-year, *n* = 10) and ‘rotating’ (7-days, *n* = 136) sites across the GAMA (Fig. S1). The data collection period excluded COVID-19 related lockdowns between March and May 2020. The fixed sites were sampled continuously for about 52 weeks, while each rotating site was sampled for a week. The ten fixed sites were selected to represent the variability in population density, socioeconomic features, and emission sources. The rotating sites were selected through a stratified random sampling method where potential measurement locations were randomly distributed across land-use strata (peri-urban, commercial/business/industrial, low-density, and high-density residential) with greater emphasis on the more urbanized AMA (Clark et al., 2020). The combination of ‘fixed’ and ‘rotating’ allowed us to utilize a finite number of monitors while capturing data across the entire geographical extent of the study area.

The quality assurance/quality control (QA/QC) procedures for PM_2.5_ measurement are described in detail elsewhere (Alli et al., 2021). Briefly, weekly gravimetric PM_2.5_ was measured using the Ultrasonic Personal Aerosol Samplers (UPAS) (Access Sensor Technologies, Fort Collins, USA) set at a flow rate of 1 liter per minute (lpm). Continuous PM_2.5_ was sampled at 1-minute intervals with the Zefan (http://www.tjzfsk.com/) real-time light-scattering based monitor that used plantower sensor and assembled in China. Monitors were housed in protective cases attached to metal poles at an average height of 4 m (±1 m). To estimate BC concentrations, the absorption coefficient (light absorbance) (10^−5^ m^−1^) of the post-weighed PM_2.5_ filters was analyzed with an image-based reflectance method that was highly correlated (*r* = 0.99) with elemental carbon concentrations on sampled filters (1 absorbance unit [1 × 10^−5^ m^−1^] is equivalent to 1.67 μg/m^3^ elemental carbon) (Jeronimo et al., 2020; Shupler et al., 2020).

We collected 654 weekly-integrated PM_2.5_ samples (518 fixed and 136 rotating sites) spanning non-Harmattan (March–October) and Harmattan seasons. Samples were included in the statistical analysis if the monitors operated for ≥75 % of the 7-day measurement period and maintained an average flow rate of ±10 % of the intended rate. When gravimetric monitors operate for <75 % (fixed sites = 19 and rotating sites = 7; 4 % of total samples) of the measurement period, they were replaced by the co-located continuous PM_2.5_ concentrations, following corrections using a correction factor (CF) derived from all co-located gravimetric vs continuous samples that met our inclusion criteria. In brief, CF was calculated such that the average of continuous PM_2.5_ measurements was equal to the gravimetric PM_2.5_ concentration at the same location over the same 7-day measurement period. Details of the correction process are described elsewhere (Alli et al., 2021). In all, only two PM_2.5_ samples from fixed sites and three from rotating sites were excluded due to complete data loss (e.g., both gravimetric and continuous monitors malfunctioned). Thus, a total of 649 weekly PM_2.5_ samples (516 fixed and 133 rotating sites) and 623 BC (497 fixed and 126 rotating sites) samples contributed to this analysis. These data spanned both the non-Harmattan (PM_2.5_/BC = 524/503 samples) and Harmattan (PM_2.5_/BC = 125/117 samples) and served as dependent variables for the season specific models.

### 2.3 Land use regression modelling

We follow typical air pollution LUR modelling approaches by regressing measured pollutant concentrations against site-specific geospatial predictors that are potential surrogates for emission sources, dispersion processes, and green spaces (Hoek et al., 2011; Lee et al., 2017; Gebreab et al., 2015). The model is then used to predict concentrations at unmonitored locations throughout the study area (Henderson et al., 2007).

#### 2.3.1 Predictor variables

We obtained or derived spatial and temporal predictor variables that had a plausible association with the emission, dispersion, or deposition of air pollution in the urban environment. Our variable selection was guided by previous LUR models (Henderson et al., 2007; Lee et al., 2017; Saucy et al., 2018; Gebreab et al., 2015; Proietti et al., 2016; He et al., 2018; Knibbs et al., 2014) and data availability. Detailed information on the predictor variables and their sources are provided in Table 1.

**Table 1:** Description of candidate spatial predictor variables.

Variable (type)	Variable sub-categories	Spatial statistics	Source (year)
Road network (*Spatial line*)	Major roads	Sum of the length of roads within buffer (m); Euclidean and inverse distance to nearest road (m)	OpenStreetMap (2019) (OpenStreetMap contributors, 2015)
	Secondary roads		
	Minor roads		
Airport (*Spatial polygon*)	NA	Euclidean distance (m) to the airport	Google Earth (2019)
Land use (*Raster*)	Commercial/business/industrial (CBI)	Total area within buffer (m^2^)	World Bank (2014) 20 m × 20 m from spot 5 imagery (World Bank, 2014)
	Informal residential areas		
	Formal residential areas		
	Other (non-built-up areas e.g., vegetation, water)		
Normalized Difference Vegetation Index (NDVI) (*Raster*)^a^	NA	Average NDVI value within buffer	United States geological survey (USGS) (2020) – 30 m × 30 m Landsat 8 imagery (U.S. Geological Survey, 2020)
Building footprints (*Spatial point*)	NA	Count within buffer	Maxar/Ecopia.ai (2020)
Rivers and waterways (*Spatial line*)	NA	Sum within buffer (m)	OpenStreetMap (2019) (OpenStreetMap contributors, 2015)
Elevation (*Raster*)	NA	NA	USGS Digital Elevation Model (DEM) (2017) (~90 m) (Verdin, 2017)
Population density within census enumeration areas (EA)^b^ (*Spatial polygon*)	NA	Average population per km^2^ within buffer	Ghana census (2010) (GSS, 2012)
Locations of human activity (S*patial point*)	Bus stations	Count within buffer; Presence within buffer;	Google Places (2020)
	Bus terminals		
	Restaurants		
	Shopping centers		

Time-related predictors			
Meteorological parameters	Temperature (°C)	Average weekly value; Presence of rainfall during the week	Measurement campaign (Clark et al., 2020); Ghana Meteorological Agency (GMA) (2020); National Oceanic and Atmospheric Administration (NOAA) (2020)
	Relative humidity (%)		
	Wind speed (m/s)		
	Wind direction		
	Rainfall		
	Mixing layer depth (m)		
	Water vapor mixing ratio		
	Solar radiation		
Month of year	January–December	NA	NA

Table shows all potential predictor variables used to build the models. The final models include a subset of these predictors chosen during the model selection process. Four circular buffer sizes with radii of 50 m, 100 m, 200 m, and 500 m were considered. NA: not applicable.

a The NDVI was calculated from spectral bands of green vegetation in Landsat 8 satellite images with the least amount of cloud cover (0.02 %) at the midpoint of the measurement campaign (January 2nd 2020).

b An enumeration area (EA) is the small geographic unit covered by a census enumerator during the 2010 census.

#### 2.3.2 Spatial and temporal variable generation

Following previous air pollution LUR studies (Gebreab et al., 2015; Proietti et al., 2016; He et al., 2018), buffers of 50, 100, 200 and 500 m were generated around the measurement sites to take into account variation in dispersion patterns, scales of influence (local and background pollution sources) and the geographic extent of our study area. We estimated the total length of each road category, rivers/waterways; total area of each land use category; total number and area of buildings, bus stops, bus terminals, restaurants, and shopping centers; elevation above sea level; average vegetation quantified by normalized difference vegetation index (NDVI) and population density within each buffer size. Euclidean distance of each monitoring site to the airport and all road categories were also calculated (Table 1).

We measured minute-by-minute temperature, relative humidity, wind speed, and wind direction data at a peri-urban background fixed site throughout the campaign, while hourly rainfall data was obtained from the Ghana Meteorological Agency. Data on mixing layer depth, water vapor mixing ratio, and solar radiation were derived from the Global Data Assimilation System (GDAS1) and downloaded from the National Oceanic and Atmospheric Administration (NOAA) using the Hybrid Single-Particle Lagrangian Integrated Trajectory (HYSPLIT) 4 model at hourly resolution. All meteorological predictors were averaged into weekly data to correspond to the measurement data.

#### 2.3.3 Model development

We developed a linear mixed-effects LUR model to interpret the relationships between PM_2.5_ or BC with predictor variables, and to predict weekly PM_2.5_ and BC concentrations at all locations (100 m resolution) across the GAMA. A log-transformation was applied to normalize the skewed distribution of the PM_2.5_ data (Moore et al., 2007; Proietti et al., 2016; Shi et al., 2020). We included random intercepts for measurement site to account for potential unmeasured site-specific influences on pollution levels and correlation among repeated samples taken at the fixed sites over the fifty-two weeks of measurement. We also incorporated random intercepts for week of the year to account for the impact of potential seasonal influences on measured PM_2.5_ and BC concentrations.

#### 2.3.4 Variable selection

We employed a two-stage variable model selection process to create a parsimonious model and maximize the percentage of explained variability (R^2^) (Gebreab et al., 2015). First, we ranked all predictor variables by the absolute strength of their linear correlation (Pearson's *r*) with measured PM_2.5_ and BC concentrations. The buffer radius for each predictor variable that had the highest rank was selected. We excluded variables where the sign of the coefficient was inconsistent with a priori assumptions (Henderson et al., 2007; Lee et al., 2017). Second, we applied a supervised forward stepwise regression procedure which allowed us to minimize the number of variables in the final models. Predictor variables selected in the first stage were added into the model starting from the variable with the highest absolute linear correlation. Selection of subsequent variables were made based on the magnitude of their added contribution to the model with a cut-off criterion of at least 1 % increase in adjusted R^2^ and *p*-value <0.05 (Saraswat et al., 2013; Lee et al., 2017; Wu et al., 2015; Miri et al., 2019). Collinearity between variables was assessed with variable inflation factor (VIF) and variables were excluded for VIF >3 (Tularam et al., 2020; Proietti et al., 2016; Amini et al., 2014). The process of variable evaluation continued until inclusion of additional variables no longer improved the model. Finally, all excluded variables were sequentially added to the models to check if an improved model could be found.

#### 2.3.5 Model evaluation

We evaluated model performance with 10-fold cross validation where models were trained with a random 90 % of samples and validated on the remaining 10 %. This procedure was repeated 10 times so that all samples were used at least once for both model training and validation (Proietti et al., 2016; Shi et al., 2020). Pearson correlation coefficients (*r* and *r*^2^) were used to compare the predicted with measured concentrations. We assessed each cross-validation technique with median absolute error (MAE), and mean error (ME) which measure random and systematic (bias) deviations in predictions, respectively. Validation results for PM_2.5_ were calculated for actual concentrations (i.e., we exponentiated the log-transformed predicted values and compared them with the measured concentrations). In addition, diagnostic plots including residual and QQ plots were used to evaluate whether the final models complied with the underlying assumptions of linear regression (Gebreab et al., 2015; Proietti et al., 2016). The models were then tested for residual spatial autocorrelation using Moran's I statistic (Abera et al., 2020; Sieber et al., 2017). Final models were applied to a regular 100 × 100 m grid covering the GAMA to generate a surface of concentrations for unmeasured locations for visualization. This spatial resolution is typical for urban LUR models (Huang et al., 2017; Bechle et al., 2015). Seasonal (non-Harmattan and Harmattan) PM_2.5_ and BC averages were estimated from the predicted weekly PM_2.5_ and BC concentrations and used to produce season-specific maps for the GAMA. Annual values were calculated as the mean of weekly concentrations from both seasons.

#### 2.3.6 Population distribution of exposure and local community socioeconomic status

We assessed inequalities in population exposure to different levels of ambient air pollution within the AMA by spatially overlapping the predicted PM_2.5_ and BC concentrations with a map of census enumeration area (EA) population distribution from Ghana's most recent national census (2010) data. An EA is the smallest geographical unit for enumeration in Ghana's national censuses. On average, EAs in AMA (Fig. S2) have a median population of 750-800 people and cover 0.03-0.04 km^2^. For each EA, we estimated annual mean PM_2.5_ and BC from the predicted seasonal concentrations and calculated number of people exposed relative to the WHO air quality guidelines for PM_2.5_.

Characterizing socio-economic disparities in air pollution exposure is vital to identifying groups/communities at highest risk of health burden and designing targeted air pollution mitigation efforts. Thus, we examined the association between measures of EA-level SES and air pollution levels in the AMA, the most urbanized and densely populated core of the GAMA. Our primary measure of neighborhood SES was the median log equivalized household consumption (Ghanaian Cedi (GH₵)) within each EA estimated from household expenditures and rent (Text S1) collected by the Ghana Living Standards Surveys (GLSS) Round 6. The GLSS data on expenditure were combined with the 2010 Ghana Population and Housing Census dataset in small area estimation models to derive relationships between estimated consumption, area, and other demographic features (Elbers et al., 2003; Corral et al., 2020) and explained in more detail in (Clark et al., 2022). We then summarized distributions of predicted PM_2.5_ and BC levels (annual and non-Harmattan means) across quintiles of EA SES (20 % of EAs in each group). Differences across groups were tested using analysis of variance and post hoc Tukey's Honest Significant Difference. Further, we examined bivariate associations between EA air pollution levels and the number of individuals with post-secondary education. All analyses were conducted in R (version 4.0.2).

## 3 Results

### 3.1 Descriptive statistics for measured PM_2.5_ and BC concentrations

The mean (SD) PM_2.5_ and BC absorbance for all weekly samples collected at the 146 monitoring sites across the GAMA were 35.1 (40.8) μg/m^3^ and 6.8 (4.3) × 10^−5^ m^−1^, respectively. PM_2.5_ exhibited substantial seasonal variations with 4-fold increase in concentrations during the Harmattan (90.3 (68.3) μg/m^3^) compared to the non-Harmattan season (21.9 (7.5) μg/m^3^). Similarly, BC levels were two times higher during the Harmattan (11.4 (5.4) × 10^−5^ m^−1^) relative to non-Harmattan season (5.7 (3.2) × 10^−5^ m^−1^). Therefore, we developed separate LUR models for Harmattan and non-Harmattan seasons to capture the observed spatiotemporal variations in pollutant concentrations.

### 3.2 LUR model performance and predictor associations

The final PM_2.5_ and BC model estimates for each season are presented in Table 2. PM_2.5_ and BC models had three to five predictors that explained 48–71 % of the semi-partial variance in the fixed effect components. For PM_2.5_, the predictors that remained in the final non-Harmattan model included negatively correlated Normalized Difference Vegetation Index (NDVI) and rainfall; and positively correlated population density (a proxy for anthropogenic sources) and road length. Conversely, only time-related variables, including relative humidity (RH), temperature, and calendar month were selected in the Harmattan PM_2.5_ model. To understand the relative importance of temporal and spatial predictors in explaining the variation in PM_2.5_, we excluded temporal variables during model selection. However, no useful Harmattan PM_2.5_ model was obtained using this method, suggesting the dominant role of meteorological variables in this season (Table S1). Like PM_2.5_, road length was an important predictor in the BC models. Further, non-Harmattan BC was negatively associated with NDVI and wind speed, while number of bus stops in an area (a proxy for traffic intensity) and RH emerged as important predictors in the Harmattan BC model.

**Table 2:** Mean associations of log PM_2.5_ and BC with spatial predictor variables in the final models^a^.

Pollutant/season	Predictors^c^ (unit)	Buffer sizes	Slope coefficient (95 % confidence interval)	Cumulative R^2^ (fixed effects)
Non-Harmattan				
PM_2.5_ (μg/m^3^)^b^ (*n* = 524 from 127 measurement sites)	Intercept	–	3.02 [2.95, 3.08]	–
	NDVI	100	−0.12 [−0.17, −0.07]	0.26
	Population density (people/km^2^)	50	0.12 [0.04, 0.20]	0.35
	Total length of major roads (m)	500	0.07 [0.01, 0.13]	0.41
	Total length of secondary roads (m)	200	0.07 [0.01, 0.13]	0.45
	Presence of rainfall	–	−0.05 [−0.09, −0.02]	0.48
BC (×10^−5^ m^−1^) (*n* = 503 from 122 measurement sites)	Intercept	–	5.17 [4.71, 5.62]	–
	Total length of major roads (m)	100	1.36 [0.89, 1.82]	0.44
	Total length of secondary roads (m)	200	0.93 [0.51, 1.34]	0.55
	NDVI	–	−0.78 [−1.08, −0.49]	0.59
	Wind speed (m/s)	–	−0.65 [−0.98, −0.31]	0.63

Harmattan				
PM_2.5_ (μg/m^3^)^b^ (*n* = 125 from 26 measurement sites)	Intercept	–	3.94 [3.69, 4.18]	–
	Relative humidity (%)	–	−0.49 [−0.59, −0.39]	0.34
	Temperature (°C)	–	0.18 [0.06, 0.29]	0.51
	Month of year: January		0.56 [0.21, 0.90]	0.69
	Month of year: February		0.52 [0.19, 0.84]	
BC (×10^−5^ m^−1^) (*n* = 117 from 24 measurement sites)	Intercept	–	9.63 [8.11, 11.16]	–
	Relative humidity (%)		−1.61 [−2.31, −0.96]	0.13
	Wind speed (m/s)	–	−1.77 [−2.57, −0.96]	0.34
	Total length of major roads (m)^c^	100	2.27 [1.17, 3.38]	0.67
	Bus stops (count)	200	2.63 [0.74, 4.57]	0.71

n: number of sites from which samples were collected for model development.

a Models included random effects for site and week of year. The direction (±) of the coefficient for predictor variables in the final models were the same as those in bivariate models (Table S2).

b PM_2.5_ concentrations were log-transformed.

c Continuous variables were standardized by subtracting the data mean and dividing by the data standard deviation. A 1-point change in a standardized variable corresponds to a 1 standard deviation increase on the original scale.

**Fig. 1:**
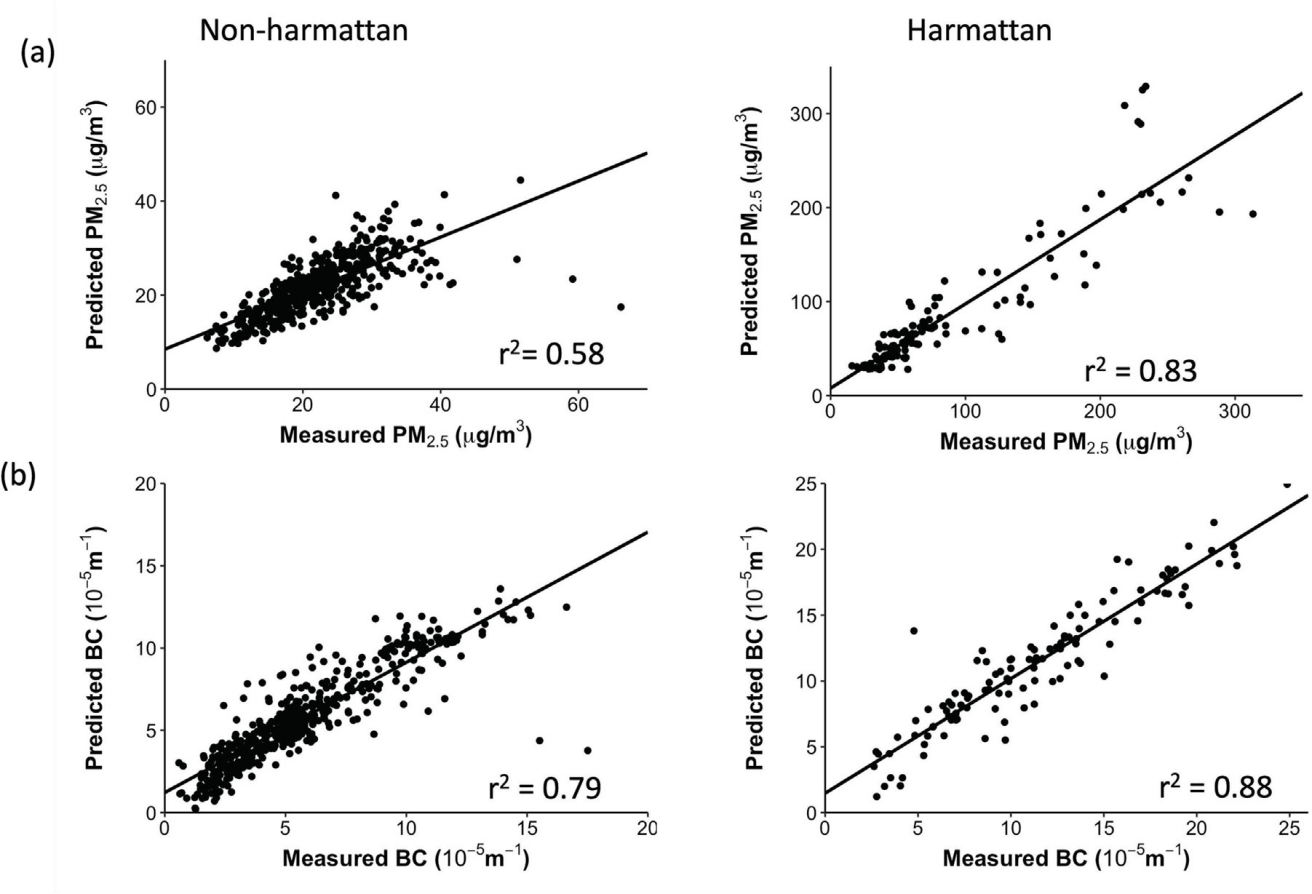
Predicted versus measured concentrations for (a) PM_2.5_ (b) BC based on the results of 10-fold cross-validation.

The cross-validation results for PM_2.5_ and BC seasonal models are shown in Table 3. For both pollutants, predicted concentrations from the seasonal models correlated strongly with measured PM_2.5_ (r: 0.76–0.91, r^2^: 0.58–0.83) and BC concentrations (r:0.89–0.94, r^2^: 0.79–0.88) (Fig. 1). Mean error (ME) for the models were close to zero indicating that systematic bias was not apparent. The range of median absolute error (MAE) for PM_2.5_ (2.05–10.63 μg/m^3^) and BC (0.65–1.09 × 10^−5^ m^−1^) models were relatively low compared with the range of measured concentrations in each season. Furthermore, Moran's I statistic of model residuals for PM_2.5_ and BC indicated that residual spatial autocorrelation was not a concern in the seasonal models. VIF values for all pollutant models were also low (<2), suggesting little to no collinearity among the variables that could inflate the coefficients.

**Table 3:** Evaluation of PM_2.5_ and BC models external generalizability with 10-fold cross validation of samples, including descriptive statistics of the measured concentrations.

Model	r	r^2^	Median absolute error	Mean error	Moran's I	Measured concentration^a^
Non-Harmattan PM_2.5_ (μg/m^3^)	0.76	0.58	2.05	0.37	−0.02	21.9 [6.1–66.2]
Harmattan PM_2.5_ (μg/m^3^)	0.91	0.83	10.47	1.35	−0.04	90.3 [15.9–313.2]
Non-Harmattan BC (×10^−5^ m^−1^)	0.89	0.79	0.65	−0.01	−0.02	5.7 [0.6–17.5]
Harmattan BC (×10^−5^ m^−1^)	0.94	0.88	1.09	−0.02	−0.09	11.4 [2.6–25.3]

a Mean [min–max].

### 3.3 Spatial and temporal variations of PM_2.5_ and BC in GAMA

**Fig. 2:**
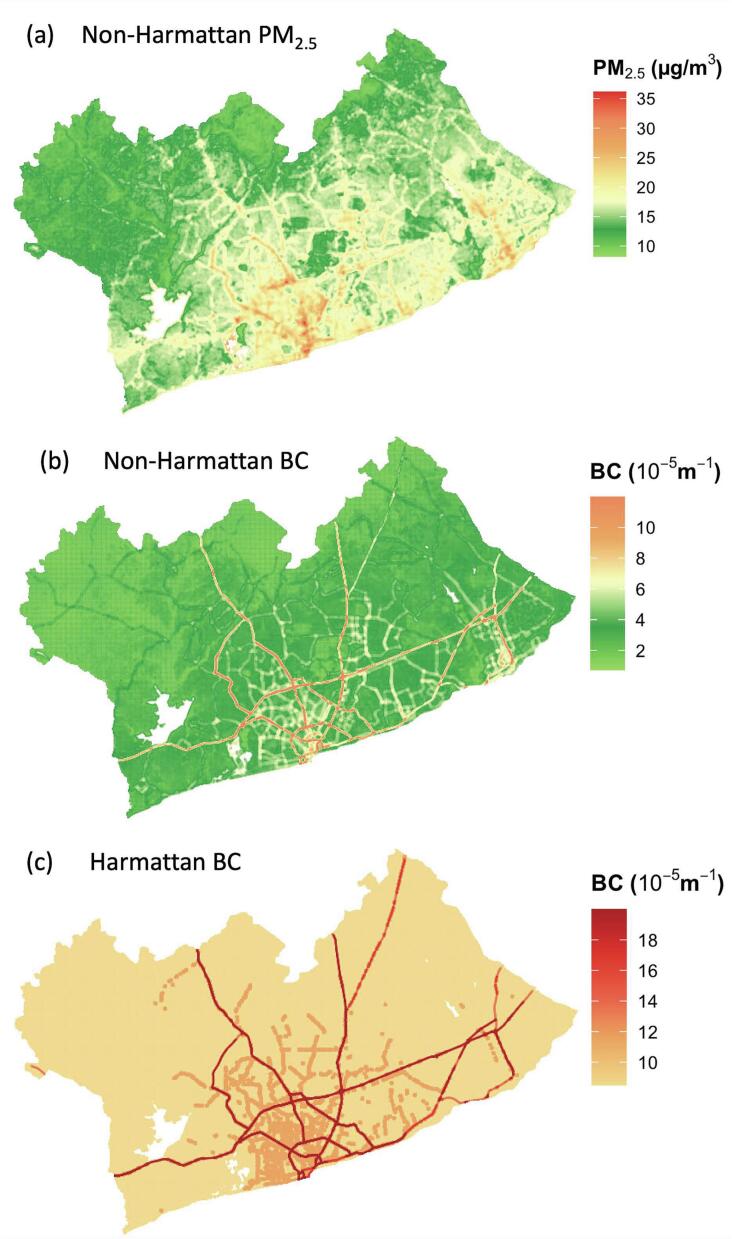
Predicted (a) Non-Harmattan PM2.5, (b) Non-Harmattan BC, and (c) Harmattan BC concentrations from the final land use regression models.

Predicted non-Harmattan PM_2.5_ concentrations were 25 % higher in the more urbanized, densely populated AMA in the south (mean: 20 μg/m^3^), than in the peri-urban districts around the north-western boundary of the GAMA (Ga-East, Ga-West, and Ga-South) (mean: 16 μg/m^3^) (Fig. 2a). The Harmattan PM_2.5_ model contained only temporal variables (Table 2); hence, the predicted value was the same for the entire metropolis (Fig. S3). However, predicted BC showed similar spatial pattern in both seasons with higher values in the city center where the road network is more extensive than in the peri-urban areas at the northwest periphery of the GAMA (Fig. 2b and c). Accordingly, predicted non-Harmattan BC concentrations were highest along major roads (mean: 9.4 × 10^−5^ m^−1^), followed by secondary roads (4.9 × 10^−5^ m^−1^) and minor roads (3.4 × 10^−5^ m^−1^). Similar trend was observed for the predicted Harmattan BC values. The seasonal pattern of predicted PM_2.5_ and BC maps were consistent with that of measured values, with 3–5-fold increase in predicted concentrations in the Harmattan (mean: PM_2.5_: 79.2 μg/m^3^, BC: 9.2 × 10^−5^ m^−1^) compared to non-Harmattan season (PM_2.5_: 15.4 μg/m^3^, BC: 3.0 × 10^−5^ m^−1^).

### 3.4 Proportion of population exposed to varying levels of air pollution in the Accra metropolis

**Fig. 3:**
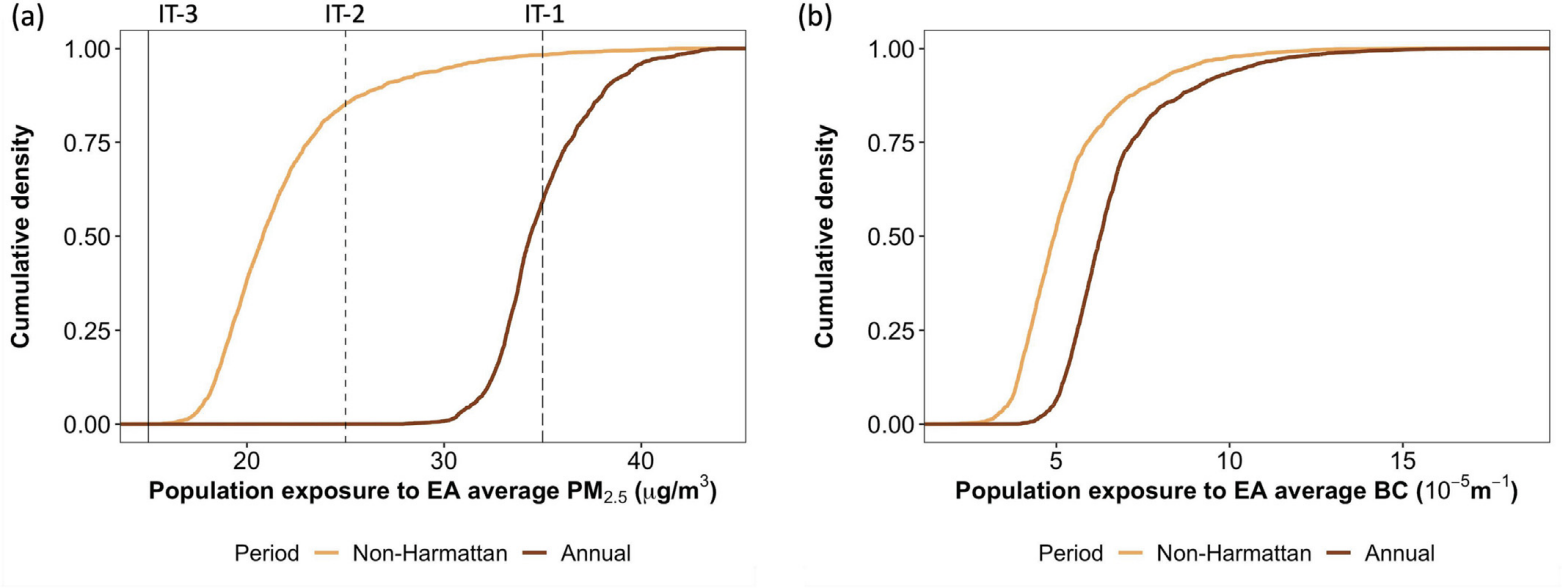
Cumulative densities of the proportion of the population in Accra Metropolitan Area (AMA) living in enumeration areas (EA) with varying (a) PM_2.5_ and (b) BC levels (population estimated from the 2010 Ghana census). IT-1: WHO Annual Average Interim Target 1 = 35 μg/m^3^; IT-2 = 25 μg/m^3^; IT-3 = 15 μg/m^3^.

All residents of AMA (~1.66 million people in 2010) lived in EAs where average predicted non-Harmattan PM_2.5_ concentrations exceeded the WHO annual guideline of 5 μg/m^3^, and even the interim target-3 (IT-3) of 15 μg/m^3^, while 15 % of AMA residents lived in EAs with values above the IT-2 of 25 μg/m^3^ (Fig. 3a). Based on the annual mean values, nearly half of the population lived in EAs with PM_2.5_ above the IT-1 of 35 μg/m^3^ (Fig. 3a). Accra residents, especially those in proximity to major and secondary roads, were exposed to high proportion of combustion by-products with 45 % and 95 % of the population residing in EAs where predicted non-Harmattan and annual mean BC values were >5 × 10^−5^ m^−1^, respectively (~8.4 μg/m^3^) (Fig. 3b).

### 3.5 Exposure inequalities by enumeration area level socioeconomic status in the Accra metropolis

**Fig. 4:**
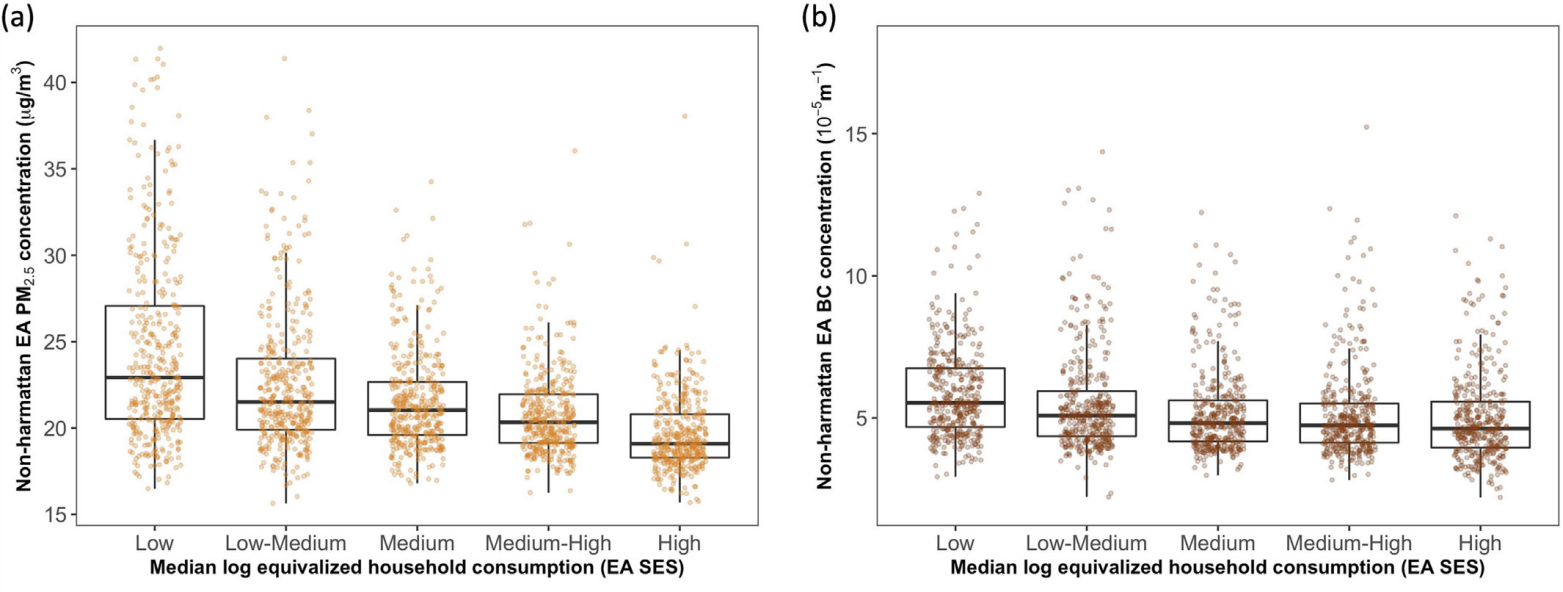
Distribution of enumeration area (EA) average non-Harmattan (a) PM_2.5_ and (b) BC concentrations across quintiles (20 % increments) of Median log equivalized household consumption (EA SES) in Accra metropolis. Median log equivalized household consumption (GH₵) was used as a proxy for EA SES. The upper and lower boundaries of the black box represent the interquartile range of the distribution and the horizontal line within the box represents the median. Each colored point is an EA average PM_2.5_ or BC concentration.

We observed a moderate inverse association between our main measure of SES (median log equivalized household consumption) and predicted air pollution levels in Accra. Predicted non-Harmattan PM_2.5_ concentrations were about 20 % higher in the poorest EAs (lower 20 % of SES distribution) compared with the wealthiest EAs (upper 20 %) (23 vs 19 μg/m^3^), with a stepwise gradient across intermediate SES quintiles (Fig. 4). We found similar trend for BC, but with a weaker association across SES groups. When education was used as a secondary metric for SES, we also observed an inverse association between the share of individuals in an EA with post-secondary education and air pollution levels (Fig. S4). The EAs in the lowest quantile of this distribution had PM_2.5_ concentrations that were 4 μg/m^3^ higher than the EAs in the highest quantile. The relative differences across SES groups remained consistent for predicted Harmattan and annual mean concentrations. The quantitative relationship between predicted air quality and SES measures are shown in Table S2.

## 4 Discussion

We leveraged 649 PM_2.5_ and 623 BC weekly integrated outdoor air pollution measurement data from 146 sites along with meteorology and land-use data to estimate in high-resolution PM_2.5_ and BC concentrations for the GAMA. Given the strong seasonal variation in air pollution, we developed separate models for the non-Harmattan (primarily local emissions) and Harmattan (local emissions enhanced by regional transport and changes in meteorology) seasons. PM_2.5_ and BC models were spatiotemporal in nature with the highest predicted values in the Harmattan season and in the city center and near major roads. Even in the non-Harmattan when concentrations are attributed primarily to local emissions, the entire population of GAMA was exposed to levels exceeding both the WHO guideline and the IT-1. Half of the residents of Accra metropolis (Ghana's capital) lived in areas with annual mean PM_2.5_ concentrations above WHO IT-3 of 35 μg/m^3^, with the highest exposures occurring in the poorest communities.

Consistent with findings from previous LUR studies in SSA cities (Saucy et al., 2018; Tularam et al., 2020; Coker et al., 2021), non-Harmattan PM_2.5_ concentrations were positively associated with population density and road length. Consequently, predicted non-Harmattan PM_2.5_ showed a distinct pattern with higher concentrations in the densely populated AMA (urban core in the south of Accra) where two-thirds of all registered vehicles in Ghana are located (Imoro Musah et al., 2020). PM_2.5_ was also negatively associated with NDVI and values were lower in the peri-urban areas along the north-western boundary, likely reflecting the effect of increased green space/vegetation in attenuating air pollution (Tularam et al., 2020). Conversely, the Harmattan PM_2.5_ model was temporal, with relative humidity, temperature and calendar month being the retained variables. The Harmattan is characterized by absence of precipitation, low wind speed, and humidity, higher temperature, lower mixing layer height, and large amounts of transported mineral dust from the Sahara Desert. This period is associated with substantial increases in PM levels as well as drastic temporal variations depending on the intensity of the dust storm episodes (Alli et al., 2021; Weinstein et al., 2010; Dionisio et al., 2010b; Baumbach et al., 1995). Thus, the overwhelming influence of these meteorology-related factors may have minimized or masked the contribution of spatial variables in the Harmattan PM_2.5_ model. Nonetheless, model performance (R^2^) for both seasons are within the overall range (0.17–0.73) for PM_2.5_ LUR models across the globe (Hoek et al., 2008) and higher than those reported in some LUR studies in SSA (Saucy et al., 2018; Abera et al., 2020).

Black carbon warms the climate (Yamineva and Liu, 2019) and is considered an important indicator of the health impacts of combustion-derived particulate matter, particularly in areas such as the GAMA where primary combustion (e.g., traffic, biomass burning) is widespread (Janssen et al., 2011). Like PM_2.5_, length of major and secondary roads, indicating traffic emissions, were the most predictive variables for BC models. This is consistent with existing BC LUR literature across the globe (Eeftens et al., 2012; Xu et al., 2021; Wang et al., 2014; Lee et al., 2015). Further, the number of bus stops in an area was positively associated with BC in the Harmattan model. This is expected as 85 % of Accra residents rely on public transportation (Kumar et al., 2004), hence, the presence of bus stops in an area can be a good proxy for traffic volume. Accordingly, the distribution of predicted BC levels in both seasons, followed road networks, with highest levels along major roads and lowest in the northwestern peri-urban areas with fewer highways. Predicted BC levels (non-Harmattan mean: ~5.3 μg/m^3^) made up a significant proportion of PM_2.5_ mass concentration (non-Harmattan mean: 16.6 μg/m^3^), reflecting results from a previous study in Accra (Zhou et al., 2013).

Like PM_2.5_, BC was higher in the Harmattan than non-Harmattan. There is a possibility that Iron content in the desert mineral dust may be contributing to small increase in absorbance levels during the dusty Harmattan period (Janssen et al., 2012). However, beside the dust, the Harmattan period is also associated with drastic changes to local meteorological conditions (Alli et al., 2021; Dionisio et al., 2010a; Wang et al., 2021). These factors are known to create slower vertical mixing and stagnant conditions that may cause progressive accumulation of local anthropogenic emissions, resulting in higher levels of not just PM, but also combustion-related pollutants like BC (Marais et al., 2014; Querol et al., 2019). Besides PM_2.5_ and BC, we also observed a similar seasonal variation in NOx levels, another combustion-related pollutant (Wang et al., 2021). Altogether, the evidence strongly points to role of local meteorology in amplifying/enhancing local air pollution beyond just dust transport during the Harmattan.

The prediction results showed that the entire GAMA had non-Harmattan PM_2.5_ concentrations that exceeded both the new (5 μg/m^3^) and previous, less ambitious (10 μg/m^3^) WHO annual guidelines. Focusing on the more urbanized city core, we estimated that half of residents in the Accra metropolis (AMA) lived in EAs where average annual PM_2.5_ concentrations were above the interim-guideline-1 (IT-1: 35 μg/m^3^). The proportions of Accra residents living in areas where PM_2.5_ levels exceed the annual WHO guideline are comparable to estimates for Asian cities (range: 98.6–100 %) (Shi et al., 2020; Long et al., 2018), but higher than those for most Latin American (Gouveia et al., 2021) and European (Sicard et al., 2021; Khomenko et al., 2021) cities (range: 58–80 %). The spatial variability of estimated BC in Accra (range: 2.1–25.5 × 10^−5^ m^−1^) was much higher than the range (0.2–5.1 × 10^−5^ m^−1^) typically found in developed countries (Hoek et al., 2011; Lee et al., 2015; Montagne et al., 2013; de Hoogh et al., 2016). Further, annual BC levels (mean: ~11.2 μg/m^3^) in Accra are several folds more than typical range of ambient levels (mean: 0.2–5.1 μg/m^3^) reported in the WHO's “good practice statement for BC” (WHO, 2021).

By SES measures, the median predicted PM_2.5_ and BC concentrations were lowest in the high-SES areas compared to low-SES areas. This inverse association was stronger for PM_2.5_ where we observed a 20 % difference in exposure between these groups. Correlation between air pollution and education, another common measure of SES yielded similar results, with lower air pollution levels in EAs with higher number of residents with post-secondary education. These findings expand previous research in four neighborhoods in Accra which showed higher PM levels in poor neighborhoods (Dionisio et al., 2010a). Our estimates are generally consistent with the widely documented disparities in air pollution exposure across SES groups (defined by income, wealth, or education) in international research (Hajat et al., 2015; Cooper et al., 2019; Bell and Ebisu, 2012). Lower SES is associated with increased susceptibility to health effects of air pollution and exacerbation of existing morbidity and mortality rates (Hajat et al., 2015; Loizeau et al., 2018; Laurent et al., 2007). Therefore, the observed differences in exposure levels signify the need to identify and address the underlying causes of exposure inequities in poorer communities in Accra. Taken together, our results for PM_2.5_ and BC add to the small but growing body of evidence (Alli et al., 2021; Zhou et al., 2013; Dionisio et al., 2010a) that improving air quality in the GAMA will require a multidimensional approach that should include environmental management programs, creation of urban green spaces, improvements to road infrastructure, support for green transportation and cleaner cooking fuels, and enforcement of existing air quality regulations. Our non-Harmattan models provide clearer guide for key emission sources that need to be included in any air quality management or policy initiatives for reducing air pollution exposure in Accra and could serve as a roadmap for other cities in the West African context.

### 4.1 Strengths and limitations

Only four PM_2.5_ LUR models have been developed for growing SSA cities, compared with hundreds in North America and Europe where air pollution has declined dramatically. The lack of monitoring in the vast majority of SSA cities has been identified as a major hindrance to mapping air pollution in this large global region that accounts for 15 % of the global population (Coker and Kizito, 2018; Abera et al., 2020). Our study addressed this limitation by using week- and year-long PM_2.5_ and BC data from 146 locations monitored during a city-wide environmental monitoring campaign (Clark et al., 2020). We are the first to develop LUR model for BC pollution in SSA. Our research improved upon previous LUR studies in SSA by leveraging high quality air pollution data and coupling spatial and temporal predictors to explain the seasonal variability in air pollutant concentrations. We showed that meteorological predictors improved model prediction performance, especially for the Harmattan season. Additionally, the spatial datasets used in this study are globally available for most major cities, thus, our approach can be readily applied to urban areas in LMICs, especially those in SSA. Furthermore, the percentage of explained variance of our models indicate that they can be applied to fill the gap as exposure estimates in health and climate impact studies in the GAMA. In particular, the spatiotemporal nature of our predictions offers the flexibility to generate PM_2.5_ and BC exposure estimates for specific periods relevant for investigating acute and chronic health outcomes. Our models could also be useful for the tracking of policies designed to improve air quality. Finally, this study explored socioeconomic disparities in air pollution exposure and provided primary evidence that low-income communities in Greater Accra bear substantial exposure burden.

Our study has some limitations to consider for future studies. Given the complex structure of the rapidly urbanizing GAMA, quantitative traffic data on local sources of pollution such as road surface material (paved or unpaved), informal industries, community biomass use, and trash/solid waste burning might improve LUR model performance. However, such datasets were not available during the study period. Another potential limitation is the temporal discrepancy between the collection of certain spatial predictors (e.g., land-use raster was created in 2014, population density and enumeration areas were derived from 2010 census) and our measurement campaign which occurred in 2019/2020. Hence, analyses of population exposure to air pollution and SES disparities using data from the most recent 2010 census were restricted to Accra where urban expansion and land-use changes are smaller, relative to other districts in the GAMA (Stow et al., 2016). Our analytical approach presumed that the spatial distribution of residents in the EAs in 2010 and 2019/2020 were similar. However, preliminary report from the 2021 census showed a 35 % increase in the population size of the Greater Accra Region (GAR), implying that the 2010 census may not perfectly reflect Accra's population characteristics for our study period. Finally, increasing the number of sites sampled during the relatively short Harmattan may improve the ability of LUR model to capture spatial patterns. Nonetheless, our results show the need for policies that are focused on multisectoral approach and equitable urban infrastructure for reducing air pollution exposure in Accra and similar settings in SSA.

### 4.2 Conclusion

Within the complex urban source-pollution and climatic environment of SSA, we used data from a large-scale measurement campaign involving systematic site selection and space-time sampling and developed high-resolution spatiotemporal PM_2.5_ and BC pollution surfaces for the Greater Accra metropolis. We used the data to assess population distribution of exposure and the role of SES in exposure disparities. Our results show the need for policies that are focused on multisectoral approach and equitable urban infrastructure for reducing air pollution exposure in Accra and similar settings in SSA.

## CRediT authorship contribution statement

**Abosede S. Alli:** Conceptualization, Data curation, Formal analysis, Methodology, Project administration, Visualization, Writing – original draft, Writing – review & editing. **Sierra N. Clark:** Data curation, Project administration, Methodology, Writing - review & editing. **Jiayuan Wang:** Data curation, Writing – review & editing. **James Bennett:** Writing – review & editing. **Allison Hughes:** Data curation, Resources, Writing – review & editing. **Majid Ezzati:** Conceptualization, Methodology, Supervision, Funding acquisition, Resources, Writing – review & editing. **Michael Brauer:** Conceptualization, Methodology, Supervision, Writing – review & editing. **James Nimo:** Data curation, Writing – review & editing. **Josephine Bedford-Moses:** Data curation, Writing – review & editing. **Solomon Baah:** Data curation, Writing – review & editing. **Alicia Cavanaugh:** Data curation, Writing - review & editing. **Samuel Agyei-Mensah:** Resources, Writing – review & editing. **George Owusu:** Writing – review & editing. **Jill Baumgartner:** Methodology, Writing – review & editing. **Raphael E. Arku:** Conceptualization, Data curation, Project administration, Supervision, Methodology, Resources, Writing - review & editing.

## Declaration of competing interest

The authors declare that they have no known competing financial interests or personal relationships that could have appeared to influence the work reported in this paper.

## Data Availability

Data will be made available on request.

## Appendix A Supplementary data

Supplementary tables and figures Image 1
