# Supplementary material for: High-resolution patterns and inequalities in ambient fine particle mass (PM2.5) and black carbon (BC) in the Greater Accra Metropolis, Ghana
Source: Sci Total Environ. 2023 Jun 1;875:162582. doi: 10.1016/j.scitotenv.2023.162582 (PMC10131145; doi:10.1016/j.scitotenv.2023.162582)
Supplement: Supplementary file 1 — Supplementary tables and figures [file mmc1.docx]

**SUPPLEMENTARY INFORMATION**

**High-resolution patterns and inequalities in ambient fine particle mass (PM_2.5_) and black carbon (BC) in the Greater Accra Metropolis, Ghana**

Abosede S Alli ^1^, Sierra N Clark ^2,3^, Jiayuan Wang ^1^, James Bennett ^2,3^, Allison Hughes ^4^, Majid Ezzati ^2,3,5,6^, Michael Brauer ^7^, James Nimo ^4^, Josephine Bedford-Moses ^4^, Solomon Baah ^4^, Alicia Cavanaugh ^8^, Samuel Agyei-Mensah ^9^, George Owusu ^10^, Jill Baumgartner ^11,12^, Raphael E Arku ^1^*

^1^ Department of Environmental Health Sciences, School of Public Health and Health Sciences, University of Massachusetts, Amherst, USA

^2^ Department of Epidemiology and Biostatistics, School of Public Health, Imperial College London, London, UK

^3^ MRC Centre for Environment and Health, School of Public Health, Imperial College London, London, UK

^4^ Department of Physics, University of Ghana, Accra, Ghana

^5^ Regional Institute for Population Studies, University of Ghana, Accra, Ghana

^6^ Abdul Latif Jameel Institute for Disease and Emergency Analytics, Imperial College London, London, UK

^7^ School of Population and Public Health, The University of British Columbia, Vancouver, Canada

^8^ Department of Geography, McGill University, Montreal, Canada

^9^ Department of Geography and Resource Development, University of Ghana, Accra, Ghana

^10^ Institute of Statistical, Social & Economic Research, University of Ghana, Accra, Ghana

^11^ Institute for Health and Social Policy, McGill University, Montreal, Canada

^12^ Department of Epidemiology, Biostatistics, and Occupational Health, McGill University, Montreal, Canada

***Corresponding author:**

Raphael E Arku**,** School of Public Health and Health Sciences, University of Massachusetts, Amherst, MA, USA**.**

E-mail: rarku@umass.edu

**Pages:** 7

**Tables:** 3

**Figures:** 3

**Text S1. List of total annual real household expenditures included in the consumption variable for SES.**^1^

Food, beverages and alcohol, tobacco, clothing and footwear, housing, electricity, water, gas and other fuels, furnishings, equipment, routine maintenance, health, transport, communications, recreation and culture, education, hotels, cafes and restaurants, and miscellaneous goods and services.

**Table S1. Harmattan model^a^ excluding temporal variables**

| Pollutant/season | Predictors^c^ (unit) | Slope coefficient (95%  confidence interval) | Cumulative R^2^ (fixed effects) |
| --- | --- | --- | --- |
| Harmattan PM_2.5_ (µg/m^3^)^b^  *(n = 127)* | Intercept | 4.20 [3.81, 4.60] | - |
|  | Distance to major roads (m) | 0.14 [0.06, 0.22] | 0.04 |

^a^ Model included random effects for site and week of year.

^b^ PM_2.5_ concentrations were log-transformed.

^c^ Continuous variables were standardized by subtracting the data mean and dividing by the data standard deviation. A 1-point change in a standardized variable corresponds to a 1 standard deviation increase on the original scale.

n: number of sites from which samples were collected for model development.

**Table S2. Bivariate mixed effect linear regression model^a^ associations between spatio-temporal predictors and PM_2.5_ and BC values.**

| **Variables^b^** | **PM_2.5_ (µg/m^3^)^c^ Coefficient [95% Confidence interval]** | | | | **BC (10^-5^m^-1^) Coefficient [95% Confidence interval]** | | | |
| --- | --- | --- | --- | --- | --- | --- | --- | --- |
|  | **Buffer** | **Non-Harmattan**  **PM_2.5_ (µg/m^3^)^c^ Coefficient [95% CI]** | **Buffer** | **Harmattan**  **PM_2.5_ (µg/m^3^)^c^ Coefficient [95% CI]** | **Buffer** | **Non-Harmattan**  **BC (10^-5^m^-1^) Coefficient [95%CI]** | **Buffer** | **Harmattan**  **BC (10^-5^m^-1^) Coefficient [95% CI]** |
| **Transportation** |  |  |  |  |  |  |  |  |
| Total length of major roads (m) | 500 | 0.15 [0.07, 0.22] | 50 | 0.02 [-0.18, 0.22] | 100 | 1.99 [1.49, 2.50] | 100 | 2.91 [1.81, 4.03] |
| Total length of secondary roads (m) | 200 | 0.13 [0.06, 0.20] | 50 | -0.05 [-0.22, 0.12] | 200 | 1.69 [1.24, 2.15] | 200 | 2.25 [0.98, 3.52] |
| Total length of minor roads (m) | 50 | -0.04 [-0.09, 0.01] | 500 | -0.08 [-0.25, 0.08] | 500 | -0.001 [-0.40, 0.40] | 200 | -1.21 [-2.70, 0.31] |
| Distance to nearest major road (m) | – | -0.08 [-0.11, -0.05] | – | 0.14 [0.06, 0.22] | – | -0.55 [-0.78, -0.31] | – | -0.82 [-1.69, 0.08] |
| Distance to nearest secondary road (m) | – | -0.08 [-0.12, -0.04] | – | 0.06 [-0.05, 0.17] | – | -0.43 [-0.73, -0.13] | – | -1.03 [-2.01, -0.05] |
| Distance to nearest minor road (m) | – | 0.06 [-0.01, 0.12] | – | 0.03 [-0.17, 0.22] | – | 1.03 [0.61, 1.46] | – | 2.41 [1.01, 3.83] |
| Distance to airport | – | -0.09 [-0.13, -0.05] | – | 0.17 [0.05, 0.29] | – | -0.74 [-1.03, -0.45] | – | -0.58 [-1.77, 0.62] |
| Presence of bus station (Yes, ref: No) | 500 | 0.08 [0.01, 0.15] | 200 | -0.03 [-0.55, 0.49] | 200 | 3.82 [1.66, 5.97] | 200 | 5.42 [1.33, 9.55] |
| Presence of bus stop (Yes, ref: No) | 500 | 0.22 [0.11, 0.32] | 500 | -0.26 [-0.57, 0.03] | 200 | 2.52 [1.75, 3.29] | 200 | 4.23 [1.97, 6.45] |
| **Land use and vegetation** |  |  |  |  |  |  |  |  |
| Total area of CBI (m^2^) | 500 | 0.09 [0.05, 0.15] | 50 | -0.06 [-0.24, 0.12] | 500 | 0.70 [0.34, 1.07] | 500 | 0.82 [-0.52, 2.15] |
| Total area of informal residential (m^2^) | 200 | 0.16 [0.09, 0.22] | 50 | -0.09 [-0.27, 0.08] | 200 | 0.89 [0.36, 1.43] | 200 | -0.60 [-2.09, 0.89] |
| Total area of formal residential (m^2^) | 100 | -0.01 [-0.07, 0.04] | 200 | -0.06 [-0.22, 0.09] | 200 | -0.22 [-0.64, 0.20] | 200 | 1.10 [-0.51, 2.74] |
| Total area of non-built-up (other) (m^2^) | 500 | -0.12 [-0.17, -0.07] | 200 | 0.12 [-0.004, 0.26] | 500 | -0.69 [-1.09, -0.29] | 500 | -0.91 [-2.19, 0.38] |
| Average NDVI (0–1) | 100 | -0.18 [-0.23, -0.14] | 100 | 0.17 [0.05, 0.30] | 100 | -1.13 [-1.48, -0.77] | 50 | -1.19 [-2.39, 0.03] |
| Total length of rivers and waterways (m) | 200 | 0.02 [-0.01, 0.05] | 200 | -0.04 [-0.25, 0.16] | 200 | 0.08 [-0.14, 0.31] | 500 | 0.96 [-0.56, 2.47] |
| **Buildings and population density** |  |  |  |  |  |  |  |  |
| Total number of buildings | 200 | 0.001 [0.001, 0.001] | 50 | -0.11 [-0.28, 0.04] | 500 | 0.76 [0.31, 1.20] | 500 | 1.18 [-0.16, 2.53] |
| Average population density (pop/km^2^) | 50 | 0.24 [0.15, 0.33] | 200 | -0.07 [-0.26, 0.11] | 500 | 0.97 [0.42, 1.52] | 500 | 1.14 [-0.42, 2.73] |
| **Locations of human activity** |  |  |  |  |  |  |  |  |
| Presence of restaurants (Yes, ref: No) | 200 | 0.17 [0.07, 0.28] | 50 | -0.03 [-0.55, 0.49] | 50 | 2.41 [1.08, 3.73] | 50 | 5.42 [1.33, 9.55] |
| Presence of shopping malls (Yes, ref: No) | 100 | 0.18 [0.001, 0.35] | 100 | -0.03 [-0.55, 0.49] | 100 | 2.28 [0.99, 3.56] | 100 | 5.42 [1.33, 9.55] |
| **Meteorological parameters** |  |  |  |  |  |  |  |  |
| Relative humidity (%) | – | -0.01 [-0.05, 0.03] | – | -0.36 [-0.46, -0.27] | – | 0.29 [-0.09, 0.66] | – | -1.73 [-2.50, -0.99] |
| Temperature (ºC) | – | 0.05 [0.003, 0.09] | – | -0.18 [-0.27, -0.08] | – | -0.16 [-0.48, 0.17] | – | -1.21 [-1.97, -0.48] |
| Presence of rainfall (Yes, ref: No) | – | -0.05 [-0.07, -0.02] | – | -0.16 [-0.48, 0.17] | – | -0.05 [-0.25, 0.15] | – | -1.21 [-3.43, 1.26] |
| Wind speed (m/s) | – | -0.04 [-0.08, -0.003] | – | -0.09 [-0.29, 0.07] | – | -0.75 [-1.05, -0.46] | – | -2.33 [-3.33, -1.32] |
| Wind direction | – | -0.01 [-0.06, 0.04] | – | 0.01 [-0.32, 0.37] |  | 0.18 [0.07, 0.29] |  | 1.89 [0.01, 3.76] |
| Mixing layer depth (m) | – | 0.0003 [-0.04, 0.04] | – | -0.18 [-0.26, -0.10] | – | -0.35 [-0.73, 0.03] | – | 0.53 [-1.13, 0.07] |
| Water vapour mixing ratio | – | -0.01 [-0.05, 0.04] | – | -0.12 [-0.27, -0.01] | – | -0.25 [-0.59, 0.09] | – | -0.96 [-1.8, -0.15] |
| Solar radiation | – | -0.01 [-0.05, 0.03] | – | 0.27 [0.15, 0.40] | – | 0.04 [-0.24, 0.31] | – | 1.27 [0.38, 2.13] |

^a^ Models included random effects for site and week of year.

^b^ Continuous variables were standardized by subtracting the data mean and dividing by the data standard deviation. A 1-point change in a standardized variable corresponds to a 1 standard deviation increase on the original scale.

^c^ PM_2.5_ concentrations were log-transformed.

**Table S3. Correlation between EA mean non-harmattan PM_2.5_ and BC and measures of EA SES.**

| **SES measure** | **PM_2.5_ (µg/m^3^)**  **r^a^ [95% CI]** | **BC (10^-5^m^-1^)**  **r^a^ [95% CI]** |
| --- | --- | --- |
| Median log equivalized household consumption | -0.38 [-0.41, -0.38] | -0.17 [-0.21, -0.12] |
| Number of individuals with post-secondary education | -0.35 [-0.38, -0.31] | -0.13 [-0.17, -0.09] |

^a^ Pearson correlation coefficient and 95% confidence intervals around correlation estimate.


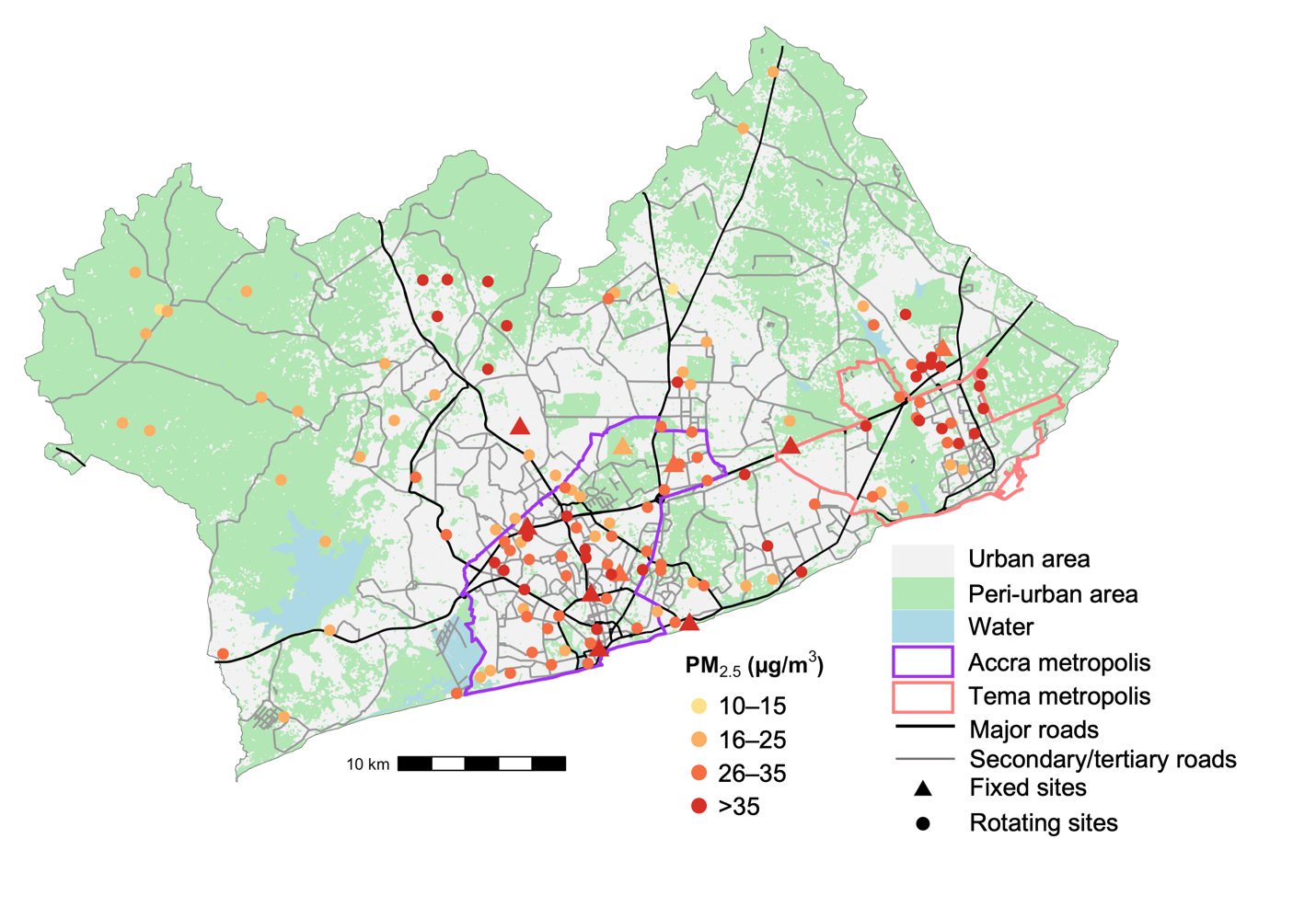
**Figure S1. Map of the study area showing land use types and distribution of the 146 monitoring sites in the GAMA.**

City boundaries are from the Ghana Statistical Service, while shapefiles for road networks and water bodies are from OpenStreetMap (2019). Land use shapefile is from World Bank (2014).


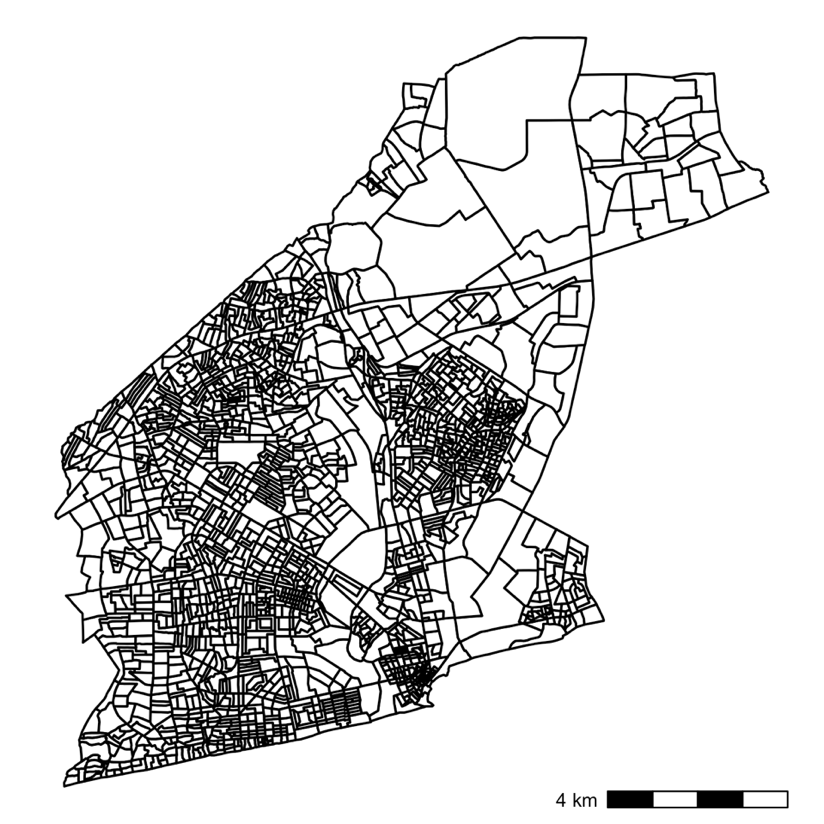


**Figure S2. Boundaries of enumeration areas in Accra metropolis for the 2010 Ghana population and housing census.** Based on this census, Accra population size was 1.66 million people.


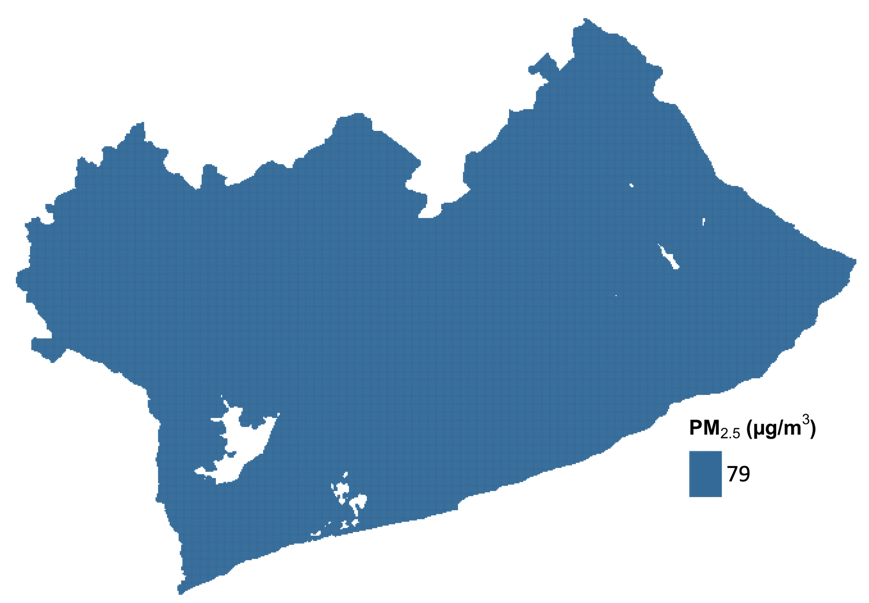


**Figure S3. Predicted Harmattan PM_2.5_ concentrations from the final land use regression model.**


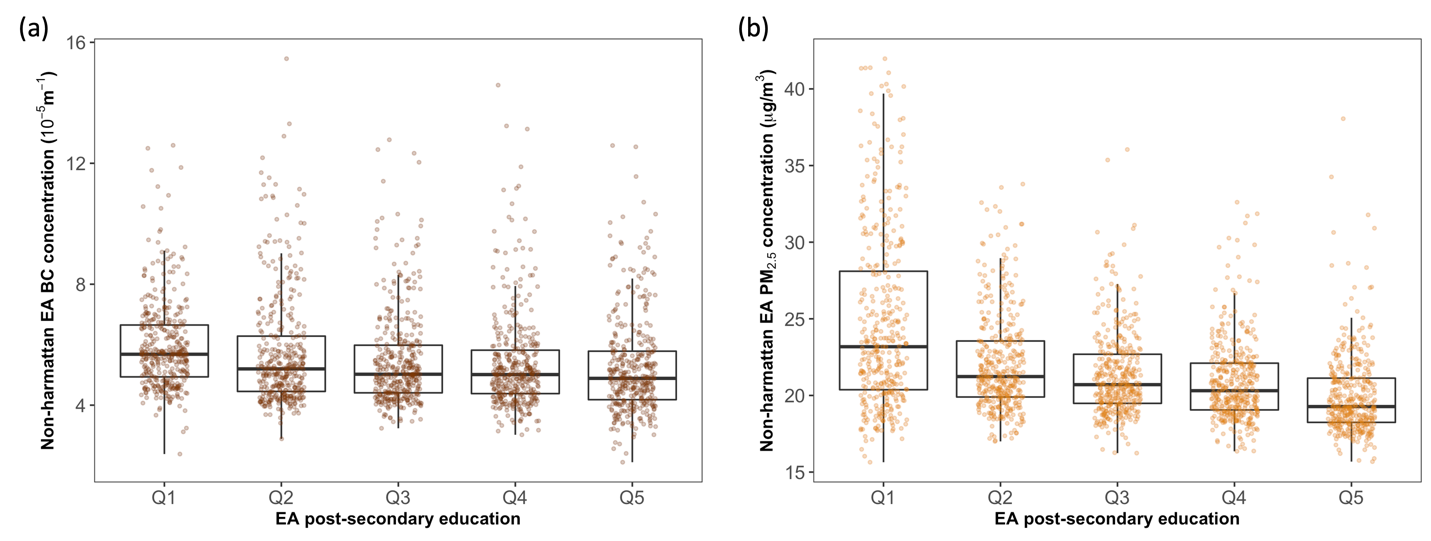


**Figure S4.** **Distribution of EA average non-harmattan (a) PM_2.5_ and (b) BC levels across quintiles (20% increments) of the number of individuals with post-secondary education in EAs in Accra metropolis.**

The black box represents the interquartile range of the distribution and the horizontal line within the box represents the median. Each point is an EA average non-harmattan PM_2.5_ or BC concentration.

**REFERENCES**

1. Cavanaugh A, Robinson BE, Bixby H, et al. Locating poverty and inequality: an application of small area estimation methods using survey and census data from Ghana (Manuscript in preparation).
